# Methemoglobinemia Following Indoxacarb Ingestion: A Unique Toxicological Presentation

**DOI:** 10.7759/cureus.59122

**Published:** 2024-04-27

**Authors:** Aniket Patel, Gajanan Chavan, Charuta Gadkari, Akhilesh Singh, Rajshree D Seram

**Affiliations:** 1 Emergency Medicine, Jawaharlal Nehru Medical College, Datta Meghe Institute of Higher Education and Research, Wardha, IND

**Keywords:** methylene blue, cyanosis, pesticide, poisoning, methemoglobinemia, indoxacarb

## Abstract

Indoxacarb, an oxadiazine insecticide, is known for its selective lethality by blocking neuronal voltage-dependent sodium channels. While primarily developed to target insect populations resistant to other pesticides, its toxicity in humans remains poorly understood. We present a case of methemoglobinemia resulting from indoxacarb ingestion, a rare manifestation of its toxic effects. A 38-year-old farmer attempted suicide by ingesting the insecticide, leading to cyanosis, hypoxemia, and characteristic arterial blood gas findings indicative of methemoglobinemia. Prompt diagnosis was challenging due to the absence of specific tests, necessitating clinical suspicion. Treatment with methylene blue and supportive therapy resulted in significant clinical improvement, highlighting the importance of early intervention in managing indoxacarb poisoning. This case underscores the need for increased awareness among healthcare providers regarding the potential toxic effects of indoxacarb. It emphasizes the importance of prompt recognition and treatment of methemoglobinemia in pesticide-related poisonings. Further research is warranted to elucidate the mechanisms underlying indoxacarb toxicity in humans and to optimize treatment strategies for affected individuals.

## Introduction

Indoxacarb, an oxadiazine insecticide, was introduced to address the challenge of insect strains developing resistance to conventional pesticides such as pyrethroids, carbamates, and organophosphates [1]. It acts selectively on insect nervous systems by blocking neuronal voltage-dependent sodium channels, resulting in paralysis and eventual death of the targeted pests [2]. Due to its specificity towards insect physiology, indoxacarb has gained popularity in agricultural settings for pest control. Despite its efficacy against insects, indoxacarb is known to exhibit low toxicity in mammals, including humans [3]. Limited data is available on the toxic effects of indoxacarb in humans, primarily due to its rare occurrence as a cause of poisoning. Reported cases often involve accidental ingestion or suicidal attempts [4]. As a result, there is a lack of comprehensive understanding regarding its clinical manifestations and management in human toxicity cases.

Methemoglobinemia, characterized by the presence of methemoglobin in the blood, is a rare but potentially serious complication associated with certain chemical exposures, including insecticide poisoning [5]. Methemoglobinemia results from the oxidation of ferrous iron (Fe2+) in hemoglobin to ferric iron (Fe3+), reducing its oxygen-carrying capacity [6]. While the exact mechanism of methemoglobinemia in indoxacarb poisoning remains unclear, it is hypothesized that metabolites of indoxacarb may interfere with the normal function of hemoglobin, leading to methemoglobin formation [7]. Prompt recognition and management of methemoglobinemia are essential to prevent complications such as tissue hypoxia and organ failure. Treatment typically involves the administration of methylene blue, which acts as a reducing agent to convert methemoglobin back to functional hemoglobin [8]. Supportive measures, including oxygen supplementation and fluid therapy, may also be necessary to stabilize the patient's condition. Given the limited literature on indoxacarb toxicity in humans and its potential to cause methemoglobinemia, we present a case report of methemoglobinemia following indoxacarb poisoning, highlighting the clinical presentation, diagnostic challenges, and successful management of this rare complication.

## Case presentation

**Figure 1:**
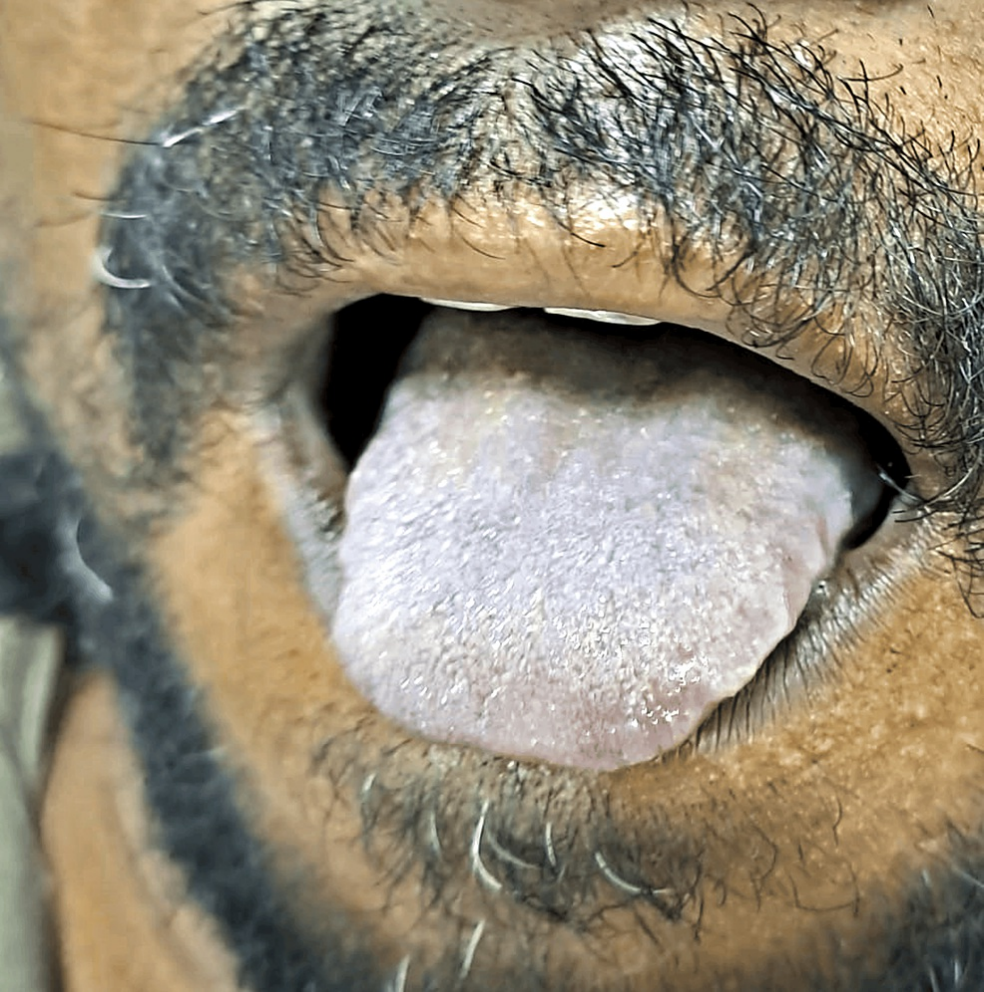
Bluish discoloration of the tongue due to central cyanosis as a result of methemoglobinemia

A 38-year-old male, identified as a farmer, presented to the emergency department following a suicide attempt by ingestion of an unidentified toxic substance. He had previously received treatment at a nearby primary healthcare facility, where he underwent atropine administration and gastric lavage. Throughout the initial assessment, his Glasgow Coma Scale score remained at 14 out of 15, and he exhibited no signs of fever. Vital signs, including pulse and blood pressure, were within normal limits. However, his oxygen saturation was 83% while receiving four liters of supplemental oxygen. Following gastric lavage, serum cholinesterase testing was conducted along with routine examination. Pupillary examination revealed symmetrical 3 mm pupils with reactive response to light assessed by automated pupillometers. Despite maintaining consciousness, the patient displayed signs of hypoventilation and exhibited evidence of tongue cyanosis, as depicted in Figure 1.

**Figure 2:**
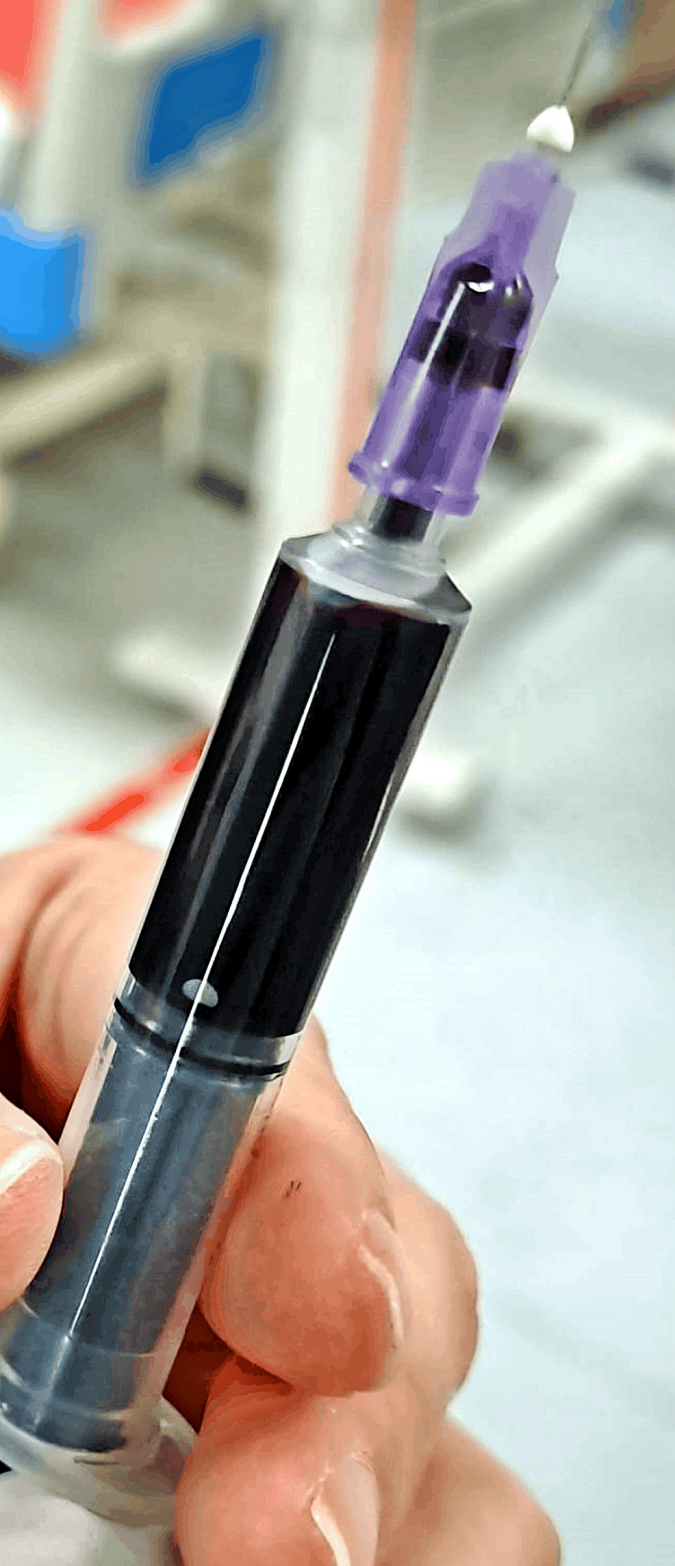
Chocolate dark brown color of arterial blood gas sample

Following a comprehensive investigation, the patient's laboratory results yielded the following findings: serum cholinesterase level was 0.76 IU/L (within the normal range of 0.6-1.5 IU/L), indicative of nominal enzymatic activity. Liver function tests revealed mildly elevated levels of alanine aminotransferase (80 IU/L) and aspartate aminotransferase (106 IU/L), suggesting potential hepatic involvement. The total leukocyte count measured 9600, within normal limits, while serum creatinine remained within the reference range. Urinalysis demonstrated a specific gravity of 1.016 with a pH of 5.8, devoid of pus or red blood cells. Notably, arterial blood gas analysis unveiled a distinct chocolate brown appearance of the patient's blood, as illustrated in Figure 2, indicative of possible methemoglobinemia.

Following oxygen analysis using a non-rebreather mask, the patient's oxygen saturation was measured at 121 mmHg. Upon further inquiry with the patient's family, they provided information regarding the ingested substance, which was identified as indoxacarb, a non-organophosphorus oxadiazine insecticide. However, due to the unavailability of serum methemoglobin testing at our facility, definitive confirmation of the diagnosis was impossible through conventional means. Nonetheless, considering the clinical presentation and suspicion of methemoglobinemia, intravenous methylene blue (1 mg/kg) was administered in 100 cc of normal saline alongside intravenous vitamin C (1 gram) and dextrose-containing fluids. Subsequently, the patient received intravenous methylene blue (60 mg) twice daily and vitamin C (500 mg) intravenously twice daily. Over the following days, the patient exhibited gradual improvement, with oxygen saturation improving to 92-95% on the second day of hospitalization. Oxygen support was tapered off gradually, improving oxygen saturation to 95-97%. By day five, the patient demonstrated remarkable gas exchange while on an oxygen face mask, with oxygen saturation reaching 98% at five liters per minute. These findings indicate the effectiveness of methylene blue and supportive therapy in managing the patient's methemoglobinemia, likely resulting from indoxacarb poisoning.

## Discussion

Indoxacarb, an oxadiazine insecticide, has gained popularity for its efficacy in controlling insect populations, particularly those resistant to other pesticides. Its mechanism of action involves blocking neuronal voltage-dependent sodium channels in insects, leading to paralysis and death [9]. While indoxacarb demonstrates low toxicity in mammals, human exposure can result in adverse effects, although documented cases are rare [10]. In our case, the patient presented with methemoglobinemia following indoxacarb ingestion, highlighting a previously undocumented complication of poisoning with this agent. Methemoglobinemia is characterized by methemoglobin, an oxidized form of hemoglobin with reduced oxygen-carrying capacity. It occurs when the ferrous (Fe2+) ion in hemoglobin is oxidized to the ferric (Fe3+) state, impairing oxygen delivery to tissues [8]. The mechanism by which indoxacarb induces methemoglobinemia remains unclear. However, it is postulated that metabolites of indoxacarb may interfere with hemoglobin function, leading to the formation of methemoglobin [11].

Diagnosis of methemoglobinemia can be challenging, particularly in cases of indoxacarb poisoning, as specific tests for indoxacarb toxicity are lacking. In our case, the diagnosis was based on clinical suspicion, supported by characteristic findings on arterial blood gas analysis, including a chocolate brown color indicative of methemoglobinemia. Additionally, the patient exhibited cyanosis of the tongue, a classic sign of methemoglobinemia [12]. Treatment of methemoglobinemia typically involves the administration of IV methylene blue, which acts as a cofactor for the enzyme nicotinamide adenine dinucleotide phosphate (NADPH)-methemoglobin reductase, facilitating the conversion of methemoglobin back to hemoglobin [5]. Supportive measures such as oxygen supplementation and fluid therapy are essential to optimize tissue oxygenation and maintain hemodynamic stability. In our case, prompt initiation of methylene blue therapy resulted in significant clinical improvement, with resolution of hypoxemia and cyanosis within days. While our case underscores the potential for methemoglobinemia as a complication of indoxacarb poisoning, it also highlights the importance of clinical vigilance and prompt intervention in managing such cases. Further research is warranted to elucidate the mechanisms underlying indoxacarb-induced methemoglobinemia and to optimize treatment strategies for affected individuals.

## Conclusions

In conclusion, our case report highlights a rare manifestation of indoxacarb poisoning, presenting as methemoglobinemia in a patient who attempted suicide by ingesting the pesticide. Despite limited documented cases of human toxicity from indoxacarb, our experience underscores the importance of considering this possibility in cases of poisoning, particularly in agricultural settings where such pesticides are commonly used. Prompt recognition and diagnosis of methemoglobinemia are crucial for initiating appropriate treatment, typically involving methylene blue administration alongside supportive measures.
